# Sympathetic nervous signaling dictates prostate cancer progression

**DOI:** 10.1038/s41420-022-00928-3

**Published:** 2022-03-10

**Authors:** D. Sigorski, E. Iżycka-Świeszewska

**Affiliations:** 1 Department of Oncology, Collegium Medicum, University of Warmia and Mazury, Al Wojska Polskiego 37, 10-228 Olsztyn, Poland; 2 Department of Oncology and Immuno-Oncology, Warmian-Masurian Cancer Center of The Ministry of The Interior and Administration’s Hospital, Olsztyn, Poland; 3 Department of Pathology and Neuropathology, Medical University of Gdańsk, Gdańsk, Poland

**Keywords:** Cancer microenvironment, Autonomic nervous system

Many different signaling pathways and biological mechanisms regulate the growth and development of cancer. The tumoral neural microenvironment and altered neurosignaling constitute a new hallmark of cancer merging as new targets for oncological therapy [1]. Nervous system involvement in tumorigenesis, cancer progression, and metastases is actively studied in prostate cancer (PCa). In the last issue of Cell Death Discovery, Dwivedy et al. showed their results of studies on adrenergic innervation and its contribution to neuroendocrine differentiation, one of the mechanisms of the castration-resistant phase of PCa (CRPC). Dwivedy et al. studied the role of β-blockers in the inhibition of neuroendocrine PCa transformation as a possible therapeutic approach (Fig. 1) (ref. [2]).

**Fig. 1:**
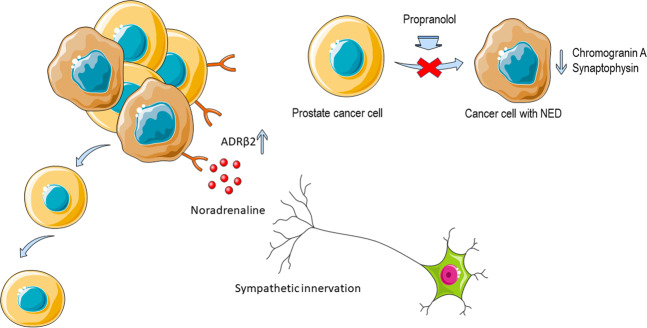
Propranolol inhibits adrenergic signaling and neuroendocrine differentiation (NED) in prostate cancer (created with smart.servier.com). ADRβ2 Adrenoceptor Beta 2.

The authors showed that sympathetic axons were present within the tumor microenvironment and interacted with neoplastic cells [2]. They found the increased innervation in half of the examined tumors, interpreting that as the evidence of active axonogenesis. The authors calculated the number of nerve fibers in the tumor and adjacent tissue, but the exact methodology including measurement area and magnification was not specified. In addition, the examined group of PCa samples was limited to only eight. Moreover, the selection criteria of the cases, as well as important data on the interval between prostatectomy and the development of metastases and castration resistance were not presented. Importantly, translation research on neuroendocrine PCa needs a large study population to get valuable results, especially since it concerns only 10% of CRPC cases. Dwivedy et al. demonstrated that sympathetic innervation was higher in some PCa than in normal adjacent tissue. The direct comparison of this research with the literature on PCa innervation/ axonogenesis is impossible, and conclusions seem to be unequivocal.

The presence of nerve branches and the intratumoral axonal network partially come from axonogenesis and neurogenesis [3]. Diverse tissue distribution of autonomic nerve fibers, creating many forms and relations with cancer cells have been reported previously [4–6]. Nerve density (ND) is a recently used marker for the assessment of innervation, but its results are difficult to compare. ND depends on the type, quantity and quality of examined tissue, calculating method, sensitivity and specificity of the used neural markers, and the individual definition of ND. Magnon et al., in their seminal ND study, showed that the overall ND was higher in high-risk PCa than in low-risk tumors [6]. Our group found that PCa innervation was higher on the proximal cancer surroundings than within PCa. Moreover, sympathetic ND was lower in PCa than in non-neoplastic prostate [5].

The authors proposed that tumors with higher sympathetic innervation were more likely to develop metastases and castration resistance, and implied that noradrenaline was the important neurotransmitter that regulated the late stage of PCa by Adrβ2. A study by Magnon et al. revealed that sympathetic innervation regulated the early phase of tumor development and the parasympathetic nervous system stimulated the late stages of PCa. They also reported that ND correlated with a poor clinical outcome [6]. Data from other studies showed the correlation between sympathetic fibers ND and biochemical failure after prostatectomy, and that adrenergic signaling reactivated dormant PCa cells in the bone marrow [7, 8]. Tumor innervation parameters may become one of the additional prognostic factors, but further studies are necessary. Studies on small core-needle biopsy material or even bigger tissue samples seem to be insufficient for the assessment of the prognostic value of ND in PCa because of tumors heterogeneity and consecutive treatment-related changes in the neoplastic population.

Finally, Dwivedi et al. reported that among adrenergic receptors, the expression of β2ADR was the most upregulated in PCa compared to normal adjacent tissue. Moreover, in vitro experiments revealed that elevated noradrenaline levels contributed to neuroendocrine differentiation in androgen-positive and negative cell lines. Thus, the disruption of neurotransmission by targeting the noradrenaline-ADRB2 axis may be a new effective therapeutic target. In this study, propranolol led to the inhibition of the morphological features of neuroendocrine differentiation, and inhibited the progression of PCa. Moreover, in the orthotopic tumor model, treatment with propranolol and castration significantly reduced tumor growth compared to castration alone or the control group [2]. Propranolol also affects cellular proliferation, apoptosis, inhibits angiogenesis, and regulates the immune system. The previous studies on PCa showed that chemical and surgical disruption of sympathetic signaling inhibits tumor growth, adrenergic nerves activate the angio-metabolic switch, and Adrβ2-cAMP-PKA cascade contributes to neuroendocrine differentiation and neurite outgrowth [6, 9–11]. Thus, the reduction of adrenergic neurotransmission by β-blockers seems to be a promising treatment option for PCa cancer [9]. Zahalka et al. found that among long-term β-blockers users, only atenolol was connected to a significant reduction in PCa risk on biopsy [12].

Despite an increasing number of studies on PCa neurobiology, the role of the autonomic nervous system, since the first study by Ayala et al., still needs further investigation. Dwivedy et al. experiments evaluating the role of β-blockers in the disruption of noradrenaline-driven neuroendocrine differentiation in PCa revealed new important data, which may change clinical attitude and therapeutic approach in the future.

## Data Availability

Data are available to the journal and the publisher upon request.
